# Quantitative sensory testing of periauricular skin in healthy adults

**DOI:** 10.1038/s41598-020-60724-w

**Published:** 2020-02-28

**Authors:** Wen Lin, Fan Zhou, Linfeng Yu, Linzhong Wan, Hua Yuan, Kelun Wang, Peter Svensson

**Affiliations:** 1 Department of Oral and Maxillofacial Surgery, Affiliated Hospital of Stomatology, Nanjing Medical University, Nanjing, China; 2 Orofacial Pain & TMD Research Unit, Institute of Stomatology, Affiliated Hospital of Stomatology, Nanjing Medical University, Nanjing, China; 3 Jiangsu Key Laboratory of Oral Diseases, Nanjing Medical University, Nanjing, China; 4 Center for Sensory-Motor Interaction (SMI), Aalborg University, Aalborg, Denmark; 5 Section of Orofacial Pain and Jaw Function, School of Dentistry and Oral Health, Aarhus University, Aarhus, Denmark; 6 Department of Dental Medicine, Karolinska Institutet, Huddinge, Sweden; 7 Scandinavian Center for Orofacial Neurosciences (SCON), Stockholm, Sweden

**Keywords:** Saliva, Oral diseases

## Abstract

The aim of this study was to investigate the test-retest reliability of quantitative sensory testing (QST) and mechanical sensitivity mapping of the periauricular skin. Twenty volunteers (10 men, 10 women) participated in two sessions at intervals of one week. Cold and warm detection threshold (CDT&WDT), cold and heat pain threshold (CPT&HPT), mechanical detection and pain threshold (MDT&MPT), pressure pain threshold (PPT) and two-point discrimination (2PD) were measured at five sites: bilateral subauricular and postauricular sites (LA, RA, LB, RB) and the dorsum of left hand (control). Pressure stimulation was applied at each of the four periauricular test sites. The test-retest reliability of the QST data implied fair to excellent agreement as evaluated by the intra-class correlation coefficients (ICC; all >0.4) for different days. There was no difference between each side in the QST parameters and mechanical sensitivity mapping (P ≥ 0.057). Significant differences between subauricular and postauricular sites were shown for WDT and PPT (P ≤ 0.028). NRS scores of mechanical sensitivity mapping showed significant effects of gender, site and point (P ≤ 0.040). QST and mechanical sensitivity mapping can be considered to be a reliable technique to assess somatosensory function of the periauricular skin.

## Introduction

The great auricular nerve (GAN) is a superficial sensory branch of the cervical plexus arising from the second and third cervical spinal nerves. It emerges from the posterior border of the sternocleidomastoid muscle (SCM) and courses anteriorly over the belly of that muscle^1,2^. Then the GAN courses superiorly and divides into 2 branches, anterior and posterior, which are both cutaneous nerves. The anterior branch supplies the skin over the parotid gland and lower preauricular region, and the posterior branch provides sensory innervation for the skin over the dorsal lower third of the auricle and mastoid process^3,4^.

Quantitative sensory testing (QST) is a reliable, noninvasive psychophysical test which allows to determine a comprehensive somatosensory profile^5^. By giving the participant quantitative somatosensory stimuli, for example sensory and pain thresholds can be measured accurately. The German Research Network on Neuropathic Pain (DFNS) has developed a standardized QST protocol which has been demonstrated to have good reliability in the orofacial region and upper and lower limbs^5,6^. Since then, more and more studies have confirmed the test-retest reliability of QST of the oral and maxillofacial region and analyzed the characteristics of the sensory and pain thresholds of the inter-oral and extra-oral regions^7,8^. However, the QST technique is limited by the need for elaboration and quantitative stimulators, professional training of operators and specialized software support, which unfortunately reduces the applicability in clinical settings. As a consequence, there remains multiple and clinically important questions related to orofacial pain and changes in somatosensory function.

Methodological advancements over the last decade have allowed new mapping techniques to be implemented in a comprehensive QST evaluation, e.g. with the repeated use of a pressure algometer for measurement of pressure pain threshold (PPT) at multiple test sites^9–11^. Compared with many of the other QST instruments, the pressure algometer is easy to use and convenient to carry, but the item it measures is relatively simple. Compared with manual palpation, pressure algometers and PPTs may consume more time, but it can provide standardized palpation pressure which is more reliable than manual palpation^12^. Recently, a study has confirmed that the technique for mechanical sensitivity mapping in the masseter muscle region and temporomandibular joint (TMJ) region with a new quantitative palpometer has an excellent reliability and can assess the spatial aspects of mechanical sensitivity in a specific anatomical region^13^.

Surgical injury and primary neurological disorders may cause GAN disturbance, so it can become necessary to perform a somatosensory examination of the periauricular skin innervated by the GAN^14–16^. However, there is so far no information on QST or mechanical sensitivity mapping of the periauricular skin. Therefore, it is not known whether the previous methods of the QST and mechanical sensitivity mapping can be applied to provide representative characteristics of the periauricular skin sensitivity.

This study aimed to investigate the test-retest reliability of QST of the periauricular skin and the surface of left hand (control), and the mechanical sensitivity mapping of the periauricular skin. An additional aim was to test for gender and site-to-site differences in the QST and mechanical sensitivity mapping of the periauricular skin.

## Materials and Methods

### Study participants

Twenty healthy young adults participated in the study. According to the gender, they were divided into two groups: 10 women (mean age ± SD: 23.2 ± 0.8 years; range: 22–25 years) and 10 men (mean age ± SD: 23.1 ± 1.1 years; range: 21–25 years). All the participants were healthy without orofacial pain complaints or symptoms of pain in head, face, and neck regions. Exclusion criteria were: history of trauma in the orofacial area that interfered with normal somatosensory function, any acute or chronic orofacial diseases (for example, burning mouth syndrome, trigeminal neuralgia, chronic headache, systemic musculoskeletal pain disorders such as fibromyalgia, or symptoms of rheumatoid arthritis, etc.), use of medication such as muscle relaxants, anticonvulsants, antidepressants, or anxiolytics within the last month, and severe systematic diseases or mental disorders. The study was conducted in accordance with the guidelines set forth in the Declaration of Helsinki II. Declaration and informed consent were obtained from all participants prior to participation. The study was approved by the Nanjing Medical University Research Ethics Committee with NO: PJ 2018-040-001.

### Experimental protocol

The experiment was performed in a quiet room with the temperature controlled between 21–25 degree Celsius. At the beginning of the experiment, all participants were informed about the purpose, content and method of this experiment, and confirmed that they fully understood. All tests were performed by the same tester using the same instrument and the tests were repeated by the same tester after one week.

**Figure 1:**
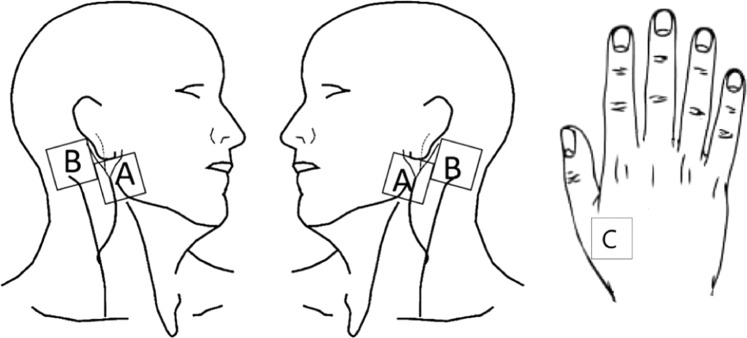
Test sites for QST: bilateral subauricular sites (**A**), bilateral postauricular sites (**B**), the surface of left hand (**C**).

The following QST parameters were tested by a method of limits: cold detection threshold (CDT), warm detection threshold (WDT), cold pain threshold (CPT), heat pain threshold (HPT), mechanical detection threshold (MDT), mechanical pain threshold (MPT), pressure pain threshold (PPT) and two-point discrimination (2PD). All parameters were measured at four sites innervated by the great auricular nerve (GAN): bilateral subauricular sites (LA, RA), bilateral postauricular sites (LB, RB) and one site innervated by the radial nerve: the dorsum of the left hand (control) (Fig. 1)^17^. In addition, a standardized palpometer (Palpeter^R^ Sunstar Suisse) with a 0.5 kg force was used to apply pressure stimulation at 9 bilateral points at each of the subauricular and postauricular sites.

### Thermal detection and thermal pain thresholds

Thermal quantitative sensory tests were performed with the use of a computerized thermal stimulator (MEDOC TSA-2001 apparatus, Medoc Ltd, Ramat-Yishai, Israel)^18,19^. The contact area of the thermode was 30 × 30 mm. CDT, WDT were measured first, followed by CPT, HPT. The temperature of the thermode started at a baseline of 32 °C and cooled down or heated up at a rate of 1 °C/s to the lower limit of 0 °C or upper limit of 55 °C. Participants were instructed to press a button on the computer mouse as soon as they perceived the thermal sensation of cold, warm, cold pain, or heat pain. Then, the procedure ended, and the temperature returned to baseline. The mean thresholds of three consecutive measurements were calculated. The verbal instructions given to the participants were in accordance with the QST guidelines as specified by the German Neuropathic Pain Network (DFNS)^5^.

### Mechanical detection and mechanical pain thresholds

MDT was measured with the use of standardized Semmes–Weinstein monofilaments with 20 different diameters (North Coast Medical, Canada). The number of each filament (1.65–6.65) corresponds to a logarithmic function of the equivalent forces of 0.008–300 g. To detect the MPT, weighted pinprick stimuli delivered with a custom-made set of seven pinprick stimulators (Aalborg University, Denmark) were used. Each stimulator had a flat contact surface of 0.2 mm that exerted forces of 8–512 mN^20^. MDT and MPT were determined by the method of limits, and defined as the geometric mean of 3 series of descending and ascending stimulus intensities^5^.

### Pressure pain threshold

A handheld pressure algometer (Algometer, MEDOC, Israel) with a probe diameter of 1.0 cm was used to test the pain sensitivity to stimuli applied to test sites by a qualified dentist who was specifically trained according to the DFNS examination protocol^5^. The algometer was applied vertically to the test sites and the applied pressure was linearly increased according to the computer prompt (30 kPa/sec). The participant was instructed to press a hold switch connected to the computer as soon as the sensation of non-painful pressure changed to a sensation of pain. The PPT value was then determined from the display. Three trials were made in random order at each test sites. There was of 1 min interval between each measurement to minimize sensitization and/or habituation to the stimulus. The mean threshold of three measurements was calculated.

### Two-point discrimination (2PD)

A vernier caliper was used to vertically contact the two tips with a distance of 15 mm to the test site, the participant was asked to judge if the stimulus was perceived as one or two points. If two points was reported, the distance between the tips was reduced by 1 mm each time until the participant reported only one point, then the threshold (distance between tips) was recorded. Three threshold measurements were made and the mean was used for further statistical analysis.

### Mechanical sensitivity mapping

A quantitative mechanical palpometer (Palpeter^R^, Sunstar Suisse SA company was used for standardized palpation in the bilateral subauricular and postauricular sites. Each of the subauricular and postauricular sites was divided into 3 × 3 grids. The palpometer with 0.5 kg force was applied to the 9 grids in randomized order^13^. All test points were stimulated for approximately 2 seconds during each measurement. After each measurement, there was a 10-second interval for the participant to rate the perceived intensity of the stimulus on a 0-50-100 numerical rating scale (NRS), in which 0 means no sensation, 50 means just barely painful, 100 means most pain imaginable^21^. In one session, both sides of the subauricular and postauricular sites were tested three times and the average NRS score of the three stimuli was calculated for each measurement point.

**Figure 2:**
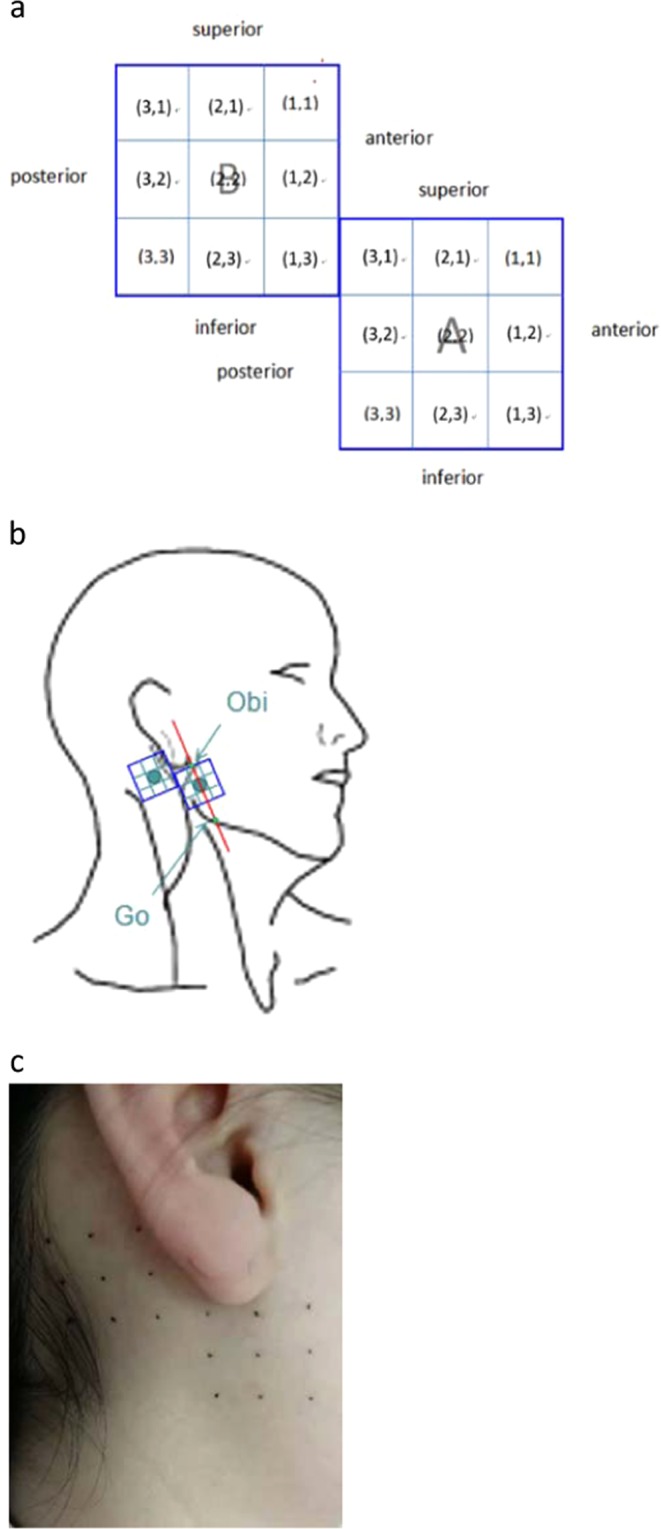
Test points for mechanical sensitivity mapping. (**a**) A standard template was fabricated to identify the test sites prior to the test, consisting of 2 squares (3*3 cm) (A,B). Each of the 2 squares was divided into 9 squares, the side of which is 10 mm. (**b**) Each of the subauricular and postauricular sites was divided into 3 × 3 grids. The center of the square A is on the line between the otobasion inferius (Obs) and the gonion (Go) and the Obi is at the midpoint of the superior edge of the square A. (**c**) The 18 points were marked on the skin of subauricular and postauricular sites with a marker pen in accordance with the standard template.

In order to make sure that the same test points were measured, a standard template was fabricated to identify the test sites prior to the test, consisting of 2 squares (3*3 cm) (A、B). Each of the 2 squares was divided into 9 squares, the side of which was 10 mm (Fig. 2a). The center of the square A is on the line between the otobasion inferius (Obi) and the gonion (Go), and the Obi is at the midpoint of the superior edge of the square A (Fig. 2b). The 18 points were marked on the skin of subauricular and postauricular sites with a marker pen in accordance with the standard template (Fig. 2c).

### Statistical analysis

All data were tested for normality distribution before further statistical analysis and log converted if not normally distributed. Intra-class correlation coefficients (ICC) and a 95% confidence interval (CI) were presented to analyze the consistency of the thresholds and NRS values over days. The value of ICC is between 0 and 1. An ICC less than 0.4 represents a poor agreement, an ICC of 0.4–0.59 is regarded fair agreement, an ICC of 0.6–0.75 is considered good agreement, and an ICC more than 0.75 indicates an excellent agreement^22^. Besides, the standard error of measurement (SEM) and its 95% confidence interval, the smallest real difference (SRD) were calculated (standard error of measurement = intra-individual standard deviation * √(1-ICC), SRD = 1.96* standard error of measurement *√2). The standard error of measurement indicates the expected error between two measurements conducted under the same circumstances in the same subject over a defined period of time. The lower the standard error of measurement, the better the test-retest-reliability^23^.

One-way analysis of variation (ANOVA) was used to test for differences in the thresholds and NRS scores over days with a significance level of 5%. Two-way ANOVA was used to test differences in the thresholds with the following factors: gender (2 levels), test sites (4 levels). Three-way ANOVA was used to test differences in NRS scores with the following factors: gender (2 levels), test sites (4 levels) and test points (9 levels).

### Ethical approval

All procedures performed in studies involving human participants were in accordance with the ethical standards of the institutional and national research committee and with the 1964 Helsinki declaration and its later amendments or comparable ethical standards.

### Informed consent

Informed consent was obtained from all individual participants included in the study.

## Results

The study was carried out from November 17, 2018 to March 18, 2019. All enrolled participants completed the experiment. All data were reported as mean and standard deviation (M ± SD).

### Reliability findings

**Figure 3:**
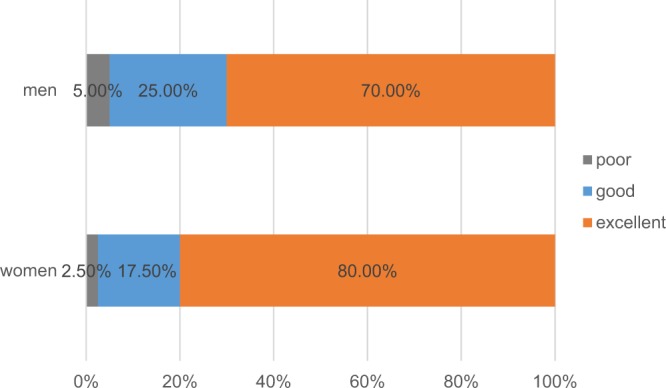
The percentages of the ICC values of each of the two groups according to their magnitude distribution.

ICC values and 95% CI of all values are listed in Tables 1 and 2 for different days. All ICC values of QST values were above 0.4 between different days (ICC_CDT_: 0.507–0.913; ICC_WDT_: 0.689–0.940; ICC_CPT_: 0.644–0.971; ICC_HPT_: 0.652–0.957; ICC_MDT_: 0.669–0.945; ICC_MPT_: 0.775–0.935; ICC_PPT_: 0.625–0.869; ICC_2PD_: 0.693–0.927) which meant the test-retest reliability of the data implied fair to excellent agreement (Table 1). Figure 3 presents the percentages of the ICC values of each of the two groups according to their magnitude distribution (Poor _women_: 2.5%; Good _women_: 17.5%; Excellent _women_: 80%; Poor _men_: 5%; Good _men_: 25%; Excellent _men_: 70%). All ICC values of NRS scores were above 0.75 between different days (ICC_LA_: 0.750–0.946; ICC_LB_: 0.776–0.947; ICC_RA_: 0.784–0.976; ICC_RB_: 0.774–0.934) which meant the test-retest reliability of the data implied excellent agreement (Table 2).

**Table 1:** The reliability scores of the QST values between different days in different sites and genders (n = 20).

item	gender	LA		LB		RA		RB		C	
		ICC (95%CI)	SEM (SRD)	ICC (95%CI)	SEM (SRD)	ICC (95%CI)	SEM (SRD)	ICC (95%CI)	SEM (SRD)	ICC (95%CI)	SEM (SRD)
CDT (°C)	women	0.761 (0.323,0.934)	0.379 (1.051)	0.913 (0.706,0.977)	0.221 (0.612)	0.874 (0.596,0.967)	0.248 (0.688)	0.893 (0.647,0.972)	0.274 (0.761)	0.507 (−0.106,0.847)	0.457 (1.268)
	men	0.834 (0.491,0.955)	0.334 (0.927)	0.550 (−0.046,0.864)	0.385 (1.066)	0.849 (0.528,0.960)	0.294 (0.814)	0.751 (0.302,0.931)	0.436 (1.208)	0.531 (−0.073,0.856)	0.364 (1.008)
WDT (°C)	women	0.940 (0.791,0.985)	0.177 (0.490)	0.855 (0.544,0.961)	0.502 (1.390)	0.899 (0.665,0.974)	0.382 (1.060)	0.827 (0.473,0.953)	0.530 (1.468)	0.850 (0.531,0.960)	0.197 (0.545)
	men	0.689 (0.179,0.911)	0.301 (0.834)	0.815 (0.443,0.950)	0.406 (1.124)	0.785 (0.373,0.941)	0.286 (0.792)	0.896 (0.657,0.973)	0.348 (0.963)	0.859 (0.555,0.962)	0.256 (0.710)
CPT (°C)	women	0.848 (0.525,0.959)	1.191 (3.301)	0.718 (0.234,0.920)	1.397 (3.873)	0.854 (0.543,0.961)	1.050 (2.910)	0.851 (0.533,0.960)	1.016 (2,817)	0.830 (0.480,0.954)	1.055 (2.925)
	men	0.644 (0.101,0.897)	0.776 (2.152)	0.971 (0.895,0.993)	1.208 (3.350)	0.833 (0.488,0.955)	1.360 (3.770)	0.935 (0.776,0.983)	1.769 (4.902)	0.848 (0.527,0.959)	0.954 (2.643)
HPT (°C)	women	0.781 (0.365,0.940)	0.830 (2.300)	0.876 (0.601,0.967)	1.149 (3.186)	0.912 (0.703,0.977)	0.917 (2.543)	0.788 (0.381,0.942)	1.114 (3.087)	0.929 (0.755,0.982)	0.656 (1.818)
	men	0.668 (0.141,0.904)	0.937 (2.596)	0.652 (0.113,0.899)	0.580 (1.608)	0.957 (0.848,0.989)	0.548 (1.519)	0.949 (0.820,0.987)	0.530 (1.470)	0.916 (0.715,0.978)	0.683 (1.892)
MDT (mN)	women	0.945 (0.806,0.986)	0.010 (0.027)	0.703 (0.206,0.916)	0.017 (0.048)	0.869 (0.580,0.965)	0.014 (0.038)	0.669 (0.144,0.905)	0.022 (0.061)	0.890 (0.640,0.971)	0.017 (0.047)
	men	0.849 (0.529,0.960)	0.020 (0.054)	0.881 (0.613,0.968)	0.014 (0.039)	0.781 (0.365,0.940)	0.013 (0.037)	0.688 (0.177,0.911)	0.016 (0.044)	0.824 (0.465,0.952)	0.065 (0.180)
MPT (mN)	women	0.885 (0.626,0.970)	0.482 (1.336)	0.917 (0.720,0.978)	0.332 (0.921)	0.893 (0.648,0.972)	0.414 (1.149)	0.935 (0.774,0.983)	0.328 (0.910)	0.848 (0.527,0.959)	0.437 (1.211)
	men	0.788 (0.38,0.942)	0.135 (0.374)	0.866 (0.572,0.964)	0.411 (1.139)	0.818 (0.452,0.951)	0.368 (1.019)	0.775 (0.352,0.938)	0.377 (1.045)	0.857 (0.549,0.962)	0.350 (0.971)
PPT (kPa)	women	0.661 (0.129,0.902)	8.972 (24.870)	0.796 (0.399,0.944)	11.621 (32.211)	0.699 (0.198,0.914)	12.320 (34.149)	0.699 (0.197,0.914)	13.512 (37.455)	0.712 (0.223,0.919)	15.056 (41.732)
	men	0.625 (0.068,0.890)	12.664 (35.102)	0.682 (0.167,0.909)	12.856 (35.635)	0.869 (0.582,0.965)	8.767 (24.300)	0.820 (0.455,0.951)	10.950 (30.352)	0.827 (0.474,0.953)	17.933 (49.708)
2PD (mm)	women	0.807 (0.425,0.947)	0.941 (2.609)	0.918 (0.721,0.979)	0.510 (1.414)	0.809 (0.430,0.948)	0.942 (2.611)	0.927 (0.748,0.981)	0.406 (1.126)	0.790 (0.386,0.943)	1.295 (3.590)
	men	0.778 (0.358,0.939)	0.323 (0.895)	0.708 (0.215,0.917)	0.475 (1.316)	0.693 (0.187,0.913)	0.377 (1.045)	0.883 (0.620,0.969)	0.229 (0.636)	0.712 (0.223,0.919)	0.680 (1.885)

Cold detection threshold (CDT), warm detection threshold (WDT), cold pain threshold (CPT), heat pain threshold (HPT), mechanical detection threshold (MDT), mechanical pain threshold (MPT), pain-pressure threshold (PPT) and two-point discrimination (2PD). LA: left subauricular site, LB: left postauricular site, RA: right subauricular site, RB: right postauricular site, C: the surface of left hand. Values are shown by ICC (95%CI), SEM and SRD.

**Table 2:** The reliability scores of the NRS scores between days in different sites and genders (n = 20).

point	gender	LA		LB		RA		RB	
		ICC (95%CI)	SEM (SRD)	ICC (95%CI)	SEM (SRD)	ICC (95%CI)	SEM (SRD)	ICC (95%CI)	SEM (SRD)
(1,1)	women	0.946 (0.811,0.986)	3.523 (9.764)	0.924 (0.740,0.980)	2.648 (7.341)	0.930 (0.760,0.982)	3.837 (10.635)	0.916 (0.716,0.978)	2.741 (7.598)
	men	0.750 (0.298,0.930)	4.258 (11.802)	0.786 (0.378,0.941)	2.359 (6.538)	0.883 (0.618,0.969)	2.671 (7.403)	0.807 (0.424,0.947)	2.815 (7.804)
(1,2)	women	0.836 (0.495,0.956)	5.990 (16.603)	0.928 (0.754,0.981)	2.436 (6.752)	0.925 (0.744,0.981)	4.272 (11.841)	0.931 (0.763,0.982)	3.073 (8.518)
	men	0.782 (0.368,0.940)	4.034 (11.181)	0.792 (0.390,0.943)	2.490 (6.901)	0.789 (0.385,0.942)	3.505 (9.715)	0.854 (0.540,0.961)	2.695 (7.469)
(1,3)	women	0.844 (0.516,0.958)	6.262 (17.358)	0.828 (0.475,0.954)	3.192 (8.847)	0.976 (0.912,0.994)	2.953 (8.185)	0.906 (0.685,0.975)	3.225 (8.94)
	men	0.811 (0.434,0.949)	2.961 (8.208)	0.790 (0.385,0.942)	2.584 (7.162)	0.791 (0.387,0.943)	4.193 (11.621)	0.868 (0.577,0.965)	2.452 (6.797)
(2,1)	women	0.867 (0.575,0.965)	4.006 (11.104)	0.906 (0.685,0.975)	3.761 (10.426)	0.945 (0.808,0.986)	2.964 (8.216)	0.875 (0.599,0.967)	3.957 (10.967)
	men	0.761 (0.323,0.934)	2.952 (8.182)	0.816 (0.445,0.950)	2.893 (8.020)	0.819 (0.453,0.951)	2.747 (7.616)	0.847 (0.524,0.959)	2.625 (7.276)
(2,2)	women	0.916 (0.715,0.978)	4.535 (12.570)	0.917 (0.719,0.978)	2.819 (7.813)	0.903 (0.677,0.975)	4.587 (12.715)	0.918 (0.722,0.979)	3.019 (8.367)
	men	0.778 (0.359,0.939)	3.425 (9.495)	0.814 (0.440,0.949)	2.144 (5.944)	0.784 (0.371,0.941)	3.384 (9.381)	0.863 (0.564,0.963)	3.006 (8.332)
(2,3)	women	0.898 (0.662,0.973)	4.836 (13.405)	0.947 (0.814,0.986)	2.393 (6.633)	0.945 (0.806,0.986)	4.103 (11.374)	0.925 (0.742,0.980)	2.945 (8.163)
	men	0.817 (0.448,0.950)	4.269 (11.832)	0.821 (0.458,0.952)	1.968 (5.454)	0.803 (0.414,0.946)	4.219 (11.695)	0.881 (0.615,0.969)	2.664 (7.385)
(3,1)	women	0.927 (0.750,0.981)	2.915 (8.081)	0.917 (0.718,0.978)	3.161 (8.762)	0.899 (0.667,0.974)	3.984 (11.043)	0.838 (0.499,0.956)	4.263 (11.816)
	men	0.852 (0.537,0.961)	2.438 (6.758)	0.848 (0.526,0.959)	1.631 (4.520)	0.795 (0.398,0.944)	2.997 (8.306)	0.774 (0.351,0.938)	4.193 (11.622)
(3,2)	women	0.904 (0.681,0.975)	4.539 (12.582)	0.883 (0.621,0.969)	3.859 (10.697)	0.933 (0.769,0.983)	3.738 (10.363)	0.825 (0.468,0.953)	4.426 (12.269)
	men	0.880 (0.610,0.968)	2.490 (6.901)	0.796 (0.400,0.944)	2.353 (6.521)	0.843 (0.512,0.958)	1.891 (5.24)	0.862 (0.561,0.963)	3.209 (8.895)
(3,3)	women	0.893 (0.647,0.972)	4.191 (11.617)	0.906 (0.686,0.975)	3.065 (8.497)	0.953 (0.833,0.988)	3.422 (9.486)	0.934 (0.771,0.983)	2.535 (7.025)
	men	0.808 (0.428,0.948)	4.189 (11.612)	0.776 (0.355,0.938)	2.167 (6.006)	0.827 (0.474,0.953)	4.409 (12.221)	0.809 (0.431,0.948)	2.871 (7.957)

LA: left subauricular site, LB: left postauricular site, RA: right subauricular site, RB: right postauricular site. Values are shown by ICC (95%CI), SEM and SRD.

The ANOVA of the values showed no significant main effects of days (P ≥ 0.132) (Table 3).

**Table 3:** Results of ANOVA analysis (F-estimates and P-values) for the QST values and NRS scores to mechanical stimuli in different sites over different days (n = 20).

	gender	LA		LB		RA		RB	
		F	P	F	P	F	P	F	P
CDT (°C)	women	1.027	0.324	0.124	0.729	0.024	0.879	0.006	0.939
	men	0.054	0.818	2.493	0.132	0.003	0.956	0.149	0.704
WDT (°C)	women	0.307	0.586	0.045	0.834	0.074	0.788	0.014	0.907
	men	0.340	0.567	0.206	0.655	0.225	0.641	0.026	0.873
CPT (°C)	women	0.491	0.492	1.205	0.287	0.006	0.939	0.111	0.743
	men	0.004	0.952	0.073	0.791	0.677	0.421	0.002	0.963
HPT (°C)	women	0.000	0.991	0.361	0.556	0.010	0.922	0.324	0.576
	men	0.640	0.434	0.026	0.874	0.125	0.728	0.025	0.875
MDT (mN)	women	0.073	0.791	0.559	0.464	0.287	0.599	1.354	0.260
	men	0.361	0.555	0.084	0.775	0.778	0.389	0.251	0.622
MPT (mN)	women	0.000	0.988	0.322	0.577	0.095	0.762	0.225	0.641
	men	0.182	0.675	0.000	0.985	0.420	0.525	0.006	0.938
PPT (kPa)	women	0.338	0.568	0.185	0.672	0.012	0.914	0.485	0.495
	men	0.038	0.848	0.024	0.878	0.277	0.605	0.157	0.697
2PD (mm)	women	0.000	1.000	0.055	0.818	0.010	0.922	0.024	0.878
	men	0.450	0.511	0.194	0.665	0.101	0.754	0.117	0.736
**point**									
(1,1)	women	0.172	0.684	0.070	0.794	0.159	0.694	0.155	0.698
	men	0.016	0.902	0.182	0.675	0.070	0.795	0.016	0.902
(1,2)	women	0.114	0.740	0.059	0.811	0.126	0.727	0.065	0.801
	men	0.565	0.462	0.693	0.416	0.125	0.728	0.222	0.643
(1,3)	women	0.004	0.948	0.216	0.648	0.048	0.829	0.000	0.995
	men	0.200	0.660	1.102	0.308	0.308	0.586	0.299	0.591
(2,1)	women	0.130	0.723	0.302	0.590	0.012	0.914	0.001	0.973
	men	0.000	0.994	0.090	0.767	0.096	0.760	0.721	0.407
(2,2)	women	0.048	0.829	0.000	0.984	0.289	0.598	0.030	0.865
	men	0.205	0.656	0.194	0.664	0.206	0.655	0.259	0.617
(2,3)	women	0.056	0.815	0.075	0.787	0.004	0.950	0.095	0.761
	men	0.030	0.865	0.004	0.948	0.301	0.590	0.094	0.763
(3,1)	women	0.002	0.966	0.137	0.716	0.321	0.578	0.023	0.880
	men	0.587	0.454	0.363	0.554	0.186	0.671	0.051	0.824
(3,2)	women	0.368	0.552	0.054	0.819	0.092	0.765	0.005	0.943
	men	0.113	0.741	0.162	0.692	0.401	0.535	0.008	0.932
(3,3)	women	0.079	0.782	0.005	0.947	0.062	0.806	0.083	0.777
	men	0.149	0.704	0.014	0.907	0.197	0.662	0.183	0.674

Cold and warm detection threshold (CDT&WDT), cold and heat pain threshold (CPT&HPT), mechanical detection and pain threshold (MDT&MPT), pain-pressure threshold (PPT) and two-point discrimination (2PD). LA: left subauricular site, LB: left postauricular site, RA: right subauricular site, RB: right postauricular site.

### Effect of gender and site

Results of ANOVA analysis (F-estimates and P-values) of gender and site effects for the QST values are listed in Table 4. Most of the QST parameters at the periauricular skin site were not gender-related except 2PD. The 2PD was significantly lower in women than in men (P = 0.048). There were no significant main effects of side in any of the QST parameters (P ≥ 0.057). Left and right sides showed corresponding values for all QST parameters and at all sites. Significant differences between the subauricular and postauricular sites were shown for WDT and PPT (P ≤ 0.028). The WDT and PPT at the postauricular site were significantly higher than at the subauricular site (P ≤ 0.028).

**Table 4:** Results of ANOVA analysis (F-estimates and P-values) of gender and site effects for the QST values.

	gender		site		LA-LB	LA-RA	LB-RB	RA-RB
	F	P	F	P	p	p	p	p
CDT (°C)	1.126	0.292	0.388	0.762	0.609	0.500	0.437	0.348
WDT (°C)	1.315	0.255	11.430	0.000***	0.001**	0.288	0.057	<0.001***
CPT (°C)	1.113	0.295	0.118	0.949	0.802	0.782	0.970	0.625
HPT (°C)	0.060	0.808	1.357	0.263	0.126	0.369	0.751	0.339
MDT (mN)	0.290	0.592	0.404	0.750	0.937	0.343	0.987	0.394
MPT (mN)	0.544	0.463	0.202	0.894	0.507	0.718	0.516	0.729
PPT (kPa)	3.635	0.061	3.900	0.012*	0.028 *	0.967	0.778	0.012*
2PD (mm)	4.034	0.048*	0.390	0.761	0.663	0.913	0.587	0.385

Cold detection threshold (CDT), warm detection threshold (WDT), cold pain threshold (CPT), heat pain threshold (HPT), mechanical detection threshold (MDT), mechanical pain threshold (MPT), pain-pressure threshold (PPT) and two-point discrimination (2PD). LA: left subauricular site, LB: left postauricular site, RA: right subauricular site, RB: right postauricular site. *P < 0.05, **P < 0.01, ***P < 0.001.

**Figure 4:**
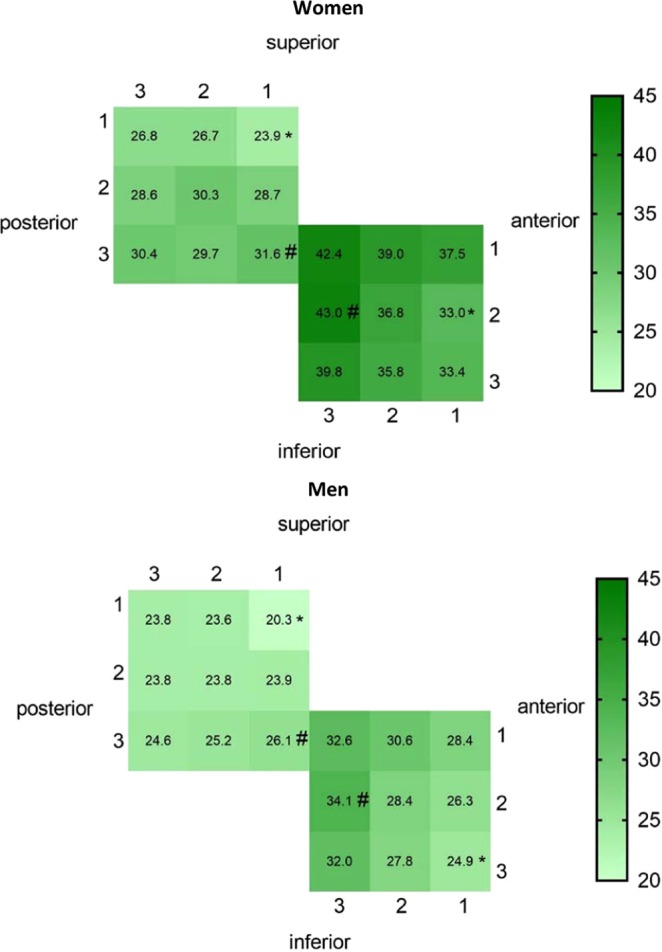
Two-dimensional illustrations of the mean NRS scores of each group at 18 points in the subauricular and postauricular sites. The more sensitive the area, the deeper the color represented. *The least sensitive point in the site. ^#^The most sensitive point in the site.

The ANOVA of the NRS scores showed that there were significant main effects of gender, site and point (Table 5). However, there were no significant main effects of side (P ≥ 0.333). Women were significantly more sensitive than men (P < 0.05). The NRS scores at the subauricular site were significantly higher than those at the postauricular site. At the subauricular site, the women reported the lowest mean NRS value (lowest sensitivity) corresponding to 33.0 at point (x,y) = (1,2) and the highest mean NRS value (highest sensitivity) 43.0 at point (x,y) = (3,2). The men reported the lowest mean NRS value as 24.9 at point (x,y) = (1,3) and the highest mean NRS value 34.1 at point (x,y) = (3,2). At the postauricular site, the women reported the lowest mean NRS value as 23.9 at point (x,y) = (1,1) and the highest mean NRS value as 31.6 at point (x,y) = (1,3). The men reported the lowest mean NRS value as 20.3 at point (x,y) = (1,1) and the highest mean NRS value as 26.1 at point (x,y) = (1,3).Two-dimensional illustrations of the mean NRS values of each of the two groups at the 18 points at the subauricular and postauricular sites are presented in Fig. 4 to show the considerable point-to-point differences in mechanical sensitivity.

**Table 5:** Results of ANOVA analysis (F-estimates and P-values) of gender, site and point effects for 0-50-100 NRS scores to mechanical stimuli.

	F	P
gender	31.252	<0.001***
site	70.879	<0.001***
point	2.039	0.040*
LA-LB		<0.001***
LA-RA		0.333
LB-RB		0.956
RA-RB		<0.001***

LA: left subauricular site, LB: left postauricular site, RA: right subauricular site, RB: right postauricular site. *P < 0.05, **P < 0.01, ***P < 0.001.

## Discussion

The present study was devoted to the development of standardized quantitative tests to assess the somatosensory function at the periauricular skin in healthy individuals. The mechanical sensitivity mapping was for the first time applied to this particular area. The results of this study showed that QST and mechanical sensitivity mapping of the periauricular skin in healthy adults are stable and with a sufficient test-retest reliability to allow clinical implementation. It was noted that, some QST parameters and mechanical sensitivity mapping were affected by gender and site and therefore needs to be taken into account in future research studies.

When the main trunk of the GAN is damaged, different degrees and different ranges of somatosensory abnormalities may appear in the skin of the lower two-thirds of the ear and periauricular region^14,15^. The somatosensory function of other sites around the external ear are dominated by three other nerves, such as the auricular branch of the vagus nerve (ABVN), the auriculotemporal nerve (ATN), and the lesser occipital nerve (LON) which were not the focus of the present study. In addition, the temporomandibular joint (TMJ) is located in the preauricular site and painful TMJ disorders may influence the somatosensory function^24,25^. Many previous studies have analyzed the QST and mechanical sensitivity mapping of the TMJ region^13,26^, so the preauricular site was not described again in this study. Though the tail of the helix and the earlobe are dominated by the GAN, these sites are too small and difficult to assess with current QST techniques. The subauricular and postauricular sites are large enough, well-positioned, and representative of the GAN innervation territory. The results of these two sites may therefore reflect the somatosensory function of the GAN to a large extent. Therefore, these two sites were assessed in this experiment.

In previous studies, the evaluation of the somatosensory function of the periauricular skin was mainly based on the stimulation with low-tech instruments such as cotton wool, a writing brush, a latex glove full of iced water, a warmed steel spoon and so on^27–29^. Such studies have used different scoring standards in order to quantify the perceived intensity of the stimulus. However, it seems difficult to ensure that the same stimulus would be applied to the same position every time, moreover, the expectation of the examiner and the psychological state of the participant may decrease the accuracy and test-retest reliability. Currently, QST is the only technology that can be used to examine the function of small nerve fibers^30^, mainly by assessment of sensory and pain thresholds. Modern physiology studies have identified three subtypes of sensory nerve fibers: Aβ, Aδ, and C fibers. A related study with the use of nerve blocks showed that CDT and MPT is transmitted by myelinated fine Aδ fibers^31^. The WDT and MDT reflect functions of unmyelinated C fibers and large, myelinated Aβ fibers, respectively^31^. The CPT is transmitted by C fibers and Aδ fibers, and the HPT is transmitted by C fibers and part of Aβ fibers^31^. Therefore, the sensory thresholds reflect dysfunctions of the peripheral nerve. In addition to the objective factors such as the method of examination, the pressure of the probe, the contact area between the probe and the skin, and the frequency of stimulation changes, the results of QST are affected by the cooperation and understanding of the participants^32^. Simultaneously, the participant´s attention and drowsiness will affect the reaction time. In general, protective measures can be taken such as indifferent stimulus (blank stimuli) and threshold changes to prevent this decline in participant-related factors.

Furthermore, a map of mechanical sensitivity was constructed in the present study with the use of a standardized palpometer in order to get more spatial details of the somatosensory function in healthy volunteers. Mapping is considered a pivotal approach in a comprehensive survey of the somatosensory system pathology^31^ and can provide an overall description of changes in somatosensory function with time or sensitivity variations among test sites^21^.

The test-retest reliability or stability of the experiment is an extremely important indicator for the evaluation and interpretation of the results which is directly related to the sensitivity and specificity of the outcome measures. In this study, a comprehensive battery included the CDT, WDT, CPT, HPT, MDT, MPT, 2PD, PPT and mechanical sensitivity mapping on the skin of left hand, bilateral subauricular sites and bilateral postauricular sites of 20 healthy Chinese in two sessions (one week apart). All ICC values of QST values were above 0.4, even 80% in the female group and 70% in the male group were above 0.75, and all ICC values of NRS scores were above 0.75, which meant the test-retest reliability of the data implied fair to excellent agreement. According to the ICC values, we believe that QST and mechanical sensitivity mapping can be used as a novel tool to evaluate the somatosensory function of the periauricular skin.

In the present study, most of the QST parameters at the periauricular skin site were not gender-related except 2PD. Women were more sensitive than men only in this particular parameter. This result is at odds with previous research. Riley *et al*.^33^ reported that women were more sensitive than men in the most of the QST parameters. The study by Komiyama^34^ shows that in the terms of QST on the cheek skin, women are much more sensitive than men. This novel finding may be caused by the different test sites, or it may be due to the relatively small sample size of this study affecting the results. In terms of the mechanical sensitivity mapping, the mean NRS score from the female group was significantly higher than that of the male group, which is consistent with previous experimental results^33,35^. Currently, the mechanism of gender-related pain threshold difference has not been fully understood. The possible factors are hormonal differences, the difference in resting blood pressure, psychological influences and the effects of dopamine and central serotonin^36,37^. In addition, when subjected to different types of external stimuli, genders will have different skin/muscle structure and thickness, anatomical characteristics, biochemical composition and different density of innervation^38^. Further research will be needed to better understand gender differences in somatosensory function.

For both QST or mechanical sensitivity mapping, there was no significant difference between the left and right sides. This suggests similar sensory thresholds on both the left and right sides, which is important information in clinical practice and future studies of patients with unilateral GAN damage.

Significant differences between the subauricular and postauricular sites were shown for WDT and PPT, which may be related to the difference in the density of nerve distribution of the branches of GAN, and may also be related to the difference in muscle and bone composition under the skin. Speaking of mechanical sensitivity mapping, the mean NRS score at the subauricular site was significantly higher than at the postauricular site. Moreover, the mechanical sensitivity was unevenly distributed over the two sites. The most sensitive part of the subauricular site was located at the midpoint of the trailing edge, and the most sensitive part of the postauricular site was at the lowest point of the leading edge. These two points are relatively close in the distance. The mean NRS value at the subauricular site showed an anterior to posterior increased inclination which may reflected in differences in somatosensory function related to the underlying parotid gland and mandible. The mean NRS value at the periauricular site showed a superior to inferior increased inclination approximately, which meant that the sensitivity of the mastoid area is lower than that of the sternocleidomastoid muscle. It could be conjectured that differences in anatomical structure lead to differences in mechanical sensitivity, which is consistent with the existing research results^13^.

The participants of the present study were 20 young healthy adults, which is a relatively small sample size, and the age effect to the QST and mechanical sensitivity was not considered. Due to the large number of the measurement parameters, it took a long time to complete the experiment, almost about 45 min, which would affect the attention and cooperation of the participants and clinical implementation. It should be considered whether it is necessary to streamline items in future measurement. Furthermore, the short-term stability of QST and mechanical sensitivity mapping was evaluated in the present study, however, its long-term stability is still not known.

## Conclusion

Both QST and mechanical sensitivity mapping are sufficiently reliable to assess the somatosensory function of the periauricular skin. The description of somatosensory function at the periauricular skin in healthy adults is a prerequisite to understand the normal range of values and to help establish a diagnosis of somatosensory dysfunction in patients undergoing surgery or following trauma to the region. In future studies, the proposed QST protocol and mechanical sensitivity mapping can be applied to patients with abnormal sensations of periauricular skin, so that it will be interesting to study further details in favor of diagnosis, treatment and prognosis in the clinic.

## Data Availability

A statistical package for social sciences version 19 (SPSS, IBM, Armonk, NY, USA) was used in this statistical procedure. The datasets generated during and/or analyzed during the current study are available from the corresponding author on reasonable request.
